# Intervention to Improve Access to Fresh Fruits and Vegetables Among Arkansas Food Pantry Clients

**DOI:** 10.5888/pcd16.180155

**Published:** 2019-01-24

**Authors:** Christopher R. Long, Brett Rowland, Pearl A. McElfish

**Affiliations:** 1 College of Medicine, University of Arkansas for Medical Sciences Northwest, Fayetteville, Arkansas; 2 Office of Community Health and Research, University of Arkansas for Medical Sciences Northwest, Fayetteville, Arkansas

## Abstract

Food pantries serve millions of Americans, yet the nutritional quality of foods distributed has been poor. Policy, systems, and environmental (PSE) changes were implemented in 3 food pantries in northwest Arkansas with the aims of improving the nutritional quality of foods distributed and increasing distribution of fresh fruits and vegetables (FFVs). Between pre-intervention and 1 year follow-up, food pantry bag audits showed increases from 20,256.38 to 25,108.46 calories distributed per household (*P* = .009) and 0.22 to 3.33 servings of FFVs distributed per person per household (*P* < .001). Findings highlight the promise of pantry-level PSE interventions.

**Summary**

**What is already known on this topic?**

Food pantries serve millions of Americans, but the nutritional quality of foods they distribute is inadequate to support a healthy diet. Food pantry clients’ fruit and vegetable consumption falls short of recommendations.

**What is added by this report?**

This study evaluates the outcomes of an intervention aimed at improving the nutritional quality of foods distributed by food pantries, documenting an increase from 0.22 to 3.33 servings of fresh fruits and vegetables distributed per person per household.

**What are the implications for public health practice?**

This report shows the promise of food pantry policy, systems, and environmental interventions to increase the servings of fresh fruits and vegetables distributed to food pantry clients.

## Objective

Approximately 5% of all US households reported using a food pantry in 2016 (1). However, the nutritional quality of pantry food is inadequate for a healthy diet (2). Likewise, pantry clients’ fruit and vegetable consumption falls short of recommendations (3). A Centers for Disease Control and Prevention Racial and Ethnic Approaches to Community Health (REACH) project sought to increase access to healthy food — including fresh fruits and vegetables (FFVs) — for pantry clients in northwest Arkansas, with particular emphasis on Pacific Islander and Hispanic clients. Evaluation of these efforts presented unique opportunities to evaluate the effectiveness of an intervention to increase access to FFVs at pantries and improve the nutrition of food distributed to clients.

## Methods

The intervention took place in 3 northwest Arkansas food pantries from September 2015 through October 2016. As part of the REACH project, these pantries were selected because they were near census tracts characterized by large proportions of Pacific Islander (up to 12.1%) and/or Hispanic residents (up to 41.4%) (4,5) compared with the populations of the 2 counties (Benton and Washington) in which the pantries were located (1.5% Pacific Islander and 16.1% Hispanic) (6,7). These pantries distributed bags of food selected by pantry staff according to each pantry’s guidelines based on food categories and client household size, affording clients minimal-to-no choice of foods.

The intervention supported pantries’ efforts to develop and implement policy, systems, and environmental (PSE) changes emphasizing 1) increased distribution of nutritious food, especially FFVs; 2) donor education about foods that support client health; and 3) improved access to healthy food for Pacific Islander and Hispanic clients. Intervention components implemented at all 3 pantries included assisting pantries with development of 1) food donation lists requesting healthier options from donors (eg, FFVs, dried beans, brown rice); 2) educational materials (eg, “The Basics of Dry Beans”) and recipes (eg, red beans and rice) in English, Spanish, and Marshallese (the primary language for most Pacific Islander clients of these pantries) to increase clients’ willingness and ability to prepare and eat healthy foods they may receive; 3) approaches to informing their donors about the donation lists (eg, via food drives or faith-based organizations); 4) approaches to displaying and distributing the educational materials and recipes to clients, with emphasis on Pacific Islander and Hispanic clients (eg, distributing English/Marshallese/Spanish cookbooks in the pantries); and 5) discussions across pantries to share ideas about sourcing healthy foods, including FFVs. To support consistent, sustained implementation of the intervention at each pantry, REACH project staff maintained regular contact with pantry staff from pre-intervention through 1 year follow-up.

A single-group pretest/posttest evaluation design was used. Measures were collected at each pantry immediately before intervention implementation and again 1 year later, minimizing variability because of seasonal FFV availability. The evaluation was ruled exempt from review by University of Arkansas for Medical Sciences’ Institutional Review Board (no. 203646).

Data collection included client surveys and pantry bag audits. Client surveys comprised demographic items administered orally in English, Spanish, or Marshallese. Survey respondent inclusion criteria included all clients aged 18 years or older visiting pantries during data collection. Each bag audit documented all food items distributed to a client household during a pantry visit and the number of members of that household. Fruits and vegetables in a fresh, perishable, unmanufactured state were categorized as FFVs. Nutrient data were captured from Nutrition Facts labels for each item. For items without Nutrition Facts labels, information was captured during data collection to describe quantities (eg, “six medium Granny Smith apples”) that were used later to generate nutrient estimates from US Department of Agriculture Food Composition Databases (8). In this way, nutrients from FFVs were incorporated into the nutritional analysis. For bags where number of household members was unknown (23.0%), missing data were replaced with mean household size from client surveys for that pantry at that point of the study (pre-intervention or follow-up). Primary outcome measures (number of FFV servings and amounts of sodium, protein, sugar, and calories distributed) were selected pre-study.

Until approximately 60 surveys and bag audits were completed per pantry at both the pre-intervention and at the follow-up, all willing, eligible clients were surveyed and every bag was audited. During each data collection visit, every eligible client was approached and invited to participate while waiting to receive food. Data collectors explained to clients that receiving food from the pantry was not contingent upon participation. No incentives were provided to clients who agreed to participate. The target sample size of 60 per pantry achieved 80% power to detect small to medium effects (*d* = .3) with independent-sample *t* tests per bag audit outcome (9).

## Results

Age, ethnicity/race, income, and total number of people per household remained similar from pre-intervention to follow-up (Table 1). However, relative to pre-intervention, a significantly greater proportion of clients at follow-up were men (36.3% vs 25.0%, *P* = .04), and there were significantly fewer children per household (1.67 vs 2.25, *P* = .02).

**Table 1:** Food Pantry Client Demographics at Pre-intervention and at One-Year Follow-up, Arkansas, 2015–2016^a^

Characteristic	Pre-intervention (n = 134)	Follow-up (n = 172)	*P* Value^b^
**Sex, no. (%)**			
Female	99 (75.0)	109 (63.7)	.04
Male	33 (25.0)	62 (36.3)	
**Age group, no. (%), y**			
18–24	7 (5.2)	6 (3.5)	.38
25–34	26 (19.4)	28 (16.4)	
35–44	30 (22.4)	27 (15.8)	
45–54	27 (20.1)	38 (22.2)	
55–64	30 (22.4)	47 (27.5)	
65–74	9 (6.7)	21 (12.3)	
≥75	5 (3.7)	4 (2.3)	
**Ethnicity/race, no. (%)**			
Hispanic alone or with any race	31 (23.1)	26 (15.4)	.30
White alone and non-Hispanic	51 (38.1)	72 (42.6)	
Pacific Islander alone and non-Hispanic	34 (25.4)	41 (24.3)	
Non-Hispanic other race(s)	18 (13.4)	30 (17.8)	
**Weekly household income, no. (%), $**			
0–614	118 (90.1)	143 (89.4)	.85
≥615	13 (9.9)	17 (10.6)	
**Household composition, mean (standard deviation)**			
Number of adults per household	2.58 (1.86)	2.55 (1.41)	.85
Number of children per household	2.25 (2.29)	1.67 (1.81)	.02
Total people per household	4.83 (3.64)	4.22 (2.74)	.10

a Numbers may not equal total because of missing data. Percentages and means are based on the number of valid responses to each item. Percentages may not total 100 due to rounding.

b *P* values for χ^2^ tests or *t* tests, based on variable type.

Calories distributed per household increased significantly from pre-intervention to follow-up (20,256.38 vs. 25,108.46, *P* = .009), although mean calories per person per household remained stable from pre-intervention to follow-up (Table 2). The mean number of servings of FFVs per person per household increased significantly from pre-intervention to follow-up (0.22 vs 3.33, *P* < .001), increasing by more than 3 servings per person per household. At pre-intervention, more than 99% of FFV servings were apples; at follow-up, FFV servings included strawberries (29.0%), tomatoes (13.6%), onions (13.5%), apples (10.6%), and others (33.2%). For each specific nutrient listed on the Nutrition Facts label, we found no significant change in mean total amounts distributed per person per household from pre-intervention to follow-up.

**Table 2:** Nutritional Analysis of Pantry Food Distributed at Pre-Intervention and at One-Year Follow-Up per Person per Household, Arkansas, 2015–2016^a^

Category	Pre-intervention, Mean (Standard Deviation) (n = 184)	Follow-up, Mean (Standard Deviation) (n = 182)	*P* Value^b^
**Per household**			
Total calories	20,256.38 (16,301.03)	25,108.46 (19,099.66)	.009
Household members	4.15 (2.25)	4.13 (2.01)	.94
**Per person per household**			
Calories	6,634.52 (6,354.98)	7,112.11 (5,469.71)	.44
Protein, g	291.02 (310.00)	274.17 (293.72)	.59
Sodium, mg	9,408.56 (8,713.17)	8,783.98 (5,800.68)	.42
Total carbohydrates, g	988.80 (939.03)	967.60 (786.77)	.82
Dietary fiber, g	124.13 (131.86)	114.64 (113.18)	.46
Sugars, g	257.45 (250.59)	250.27 (169.68)	.75
Total fat, g	233.62 (260.70)	242.51 (282.09)	.75
Saturated fat, g	61.17 (65.97)	63.14 (75.84)	.79
*Trans* fat, g	0.21 (0.93)	0.35 (1.10)	.17
Cholesterol, mg	963.68 (1048.91)	889.52 (1,327.13)	.55
Fresh fruit and vegetable servings	0.22 (1.38)	3.33 (7.69)	<.001

a Data sources for nutritional analysis include each food item’s Nutrition Facts label and, for food items that did not have Nutrition Facts labels (eg, fresh vegetables), estimates per item based on US Department of Agriculture Food Composition Databases (8). Means are based on the number of valid responses to each item.

b *P* values for *t* tests.

Table 3 characterizes the nutritional quality for each nutrient per 2,000-calorie portion of food distributed at pre-intervention and at follow-up. These are compared to the daily reference values (DRVs) based on a daily caloric intake of 2,000 calories per day for adults and children aged 4 years or older from the Food and Drug Administration’s (2016) revision of Nutrition Facts labels (10). Milligrams of sodium per 2,000 calories declined from 2,798.78 to 2,404.24, which exceeds the DRV by approximately 100 mg. Grams of protein per 2,000 calories declined from 89.40 to 78.81, which exceeds the DRV by approximately 25 g. There is no DRV for total sugars, but means at pre-intervention and at follow-up were 72 g or more of sugars per 2,000 calories, which accounts for approximately 14% of 2,000 calories (10). There were 0.08 FFV servings per 2,000 calories at pre-intervention and 0.80 at follow-up.

**Table 3:** Nutritional Analysis of Pantry Food Distributed at Pre-Intervention and at One-Year Follow-Up per 2,000 Calories of Pantry Food Distributed, Arkansas, 2015–2016^a^

Nutrient (Daily Reference Value^b^)	Pre-intervention, Mean (n = 184)	Follow-up, Mean (n = 182)	Difference Between Pre-intervention and Follow-up
Protein, g (50 g)	89.40	78.81	−10.59
Sodium, mg (2,300 mg)	2,798.78	2,404.24	−394.54
Total carbohydrates, g (275 g)	296.86	279.28	−17.58
Dietary fiber, g (28 g)	38.34	33.34	−5.00
Sugars^c^, g	75.25	72.00	−3.25
Total fat, g (78 g)	71.80	70.50	−1.30
Saturated fat, g (20 g)	18.67	18.42	−0.25
*Trans* fat^c^, g	0.05	0.10	+0.05
Cholesterol, mg (300 mg)	286.22	262.06	−24.16
Fresh fruit and vegetable servings^c^	0.08	0.80	+0.72

a Data sources for nutritional analysis include each food item’s Nutrition Facts label and, for food items that did not have Nutrition Facts labels (eg, fresh vegetables), estimates per item based on US Department of Agriculture Food Composition Databases (8). Means are based on the number of valid responses to each item.

b Daily reference value recommendations based on daily caloric intake of 2,000 calories for adults and children aged ≥4 years in Food and Drug Administration’s (2016) revision of Nutrition Facts labels (10).

c Food and Drug Administration’s (2016) revision of Nutrition Facts labels does not indicate a daily reference value recommendation (10).

## Discussion

In 3 pantries that implemented PSE changes to improve client access to healthy foods, bag audits documented a significant increase in the mean amount of FFVs distributed per person per household at follow-up. However, per 2,000 calories, the increase in FFVs was modest (from approximately zero at pre-intervention to 0.80 servings at follow-up). The amounts of specific nutrients per person remained constant from pre-intervention to follow-up, even as calories increased. Per 2,000 calories, the amount of protein was adequate at pre-intervention and at follow-up. In contrast, sodium exceeded DRV (<2,300 mg) (10) at both time points, although by only approximately 100 mg at follow-up. Given the amount of sugars distributed per 2,000 calories at both time points (75.25 g and 72.00 g) and the small amount of FFVs distributed, the number of calories from added sugars may have exceeded guidelines (<10% of calories per day) (11). However, Nutrition Facts labels did not separate added from total sugars, so a conclusive determination could not be reached.

Limitations of this study include the small number of pantries evaluated and a lack of data from nonintervention pantries. These limitations were necessary, given the time investment required to capture, process, and analyze this first-of-its-kind data set of nutrient information for food distributed to approximately 1,500 client household members from 3 pantries at 2 time points 1 year apart. With respect to improving access to healthy foods for Pacific Islander and Hispanic clients, all clients of these pantries benefited from improvements in FFV distribution and nutritional quality. However, we saw no change in proportions of clients identifying as Pacific Islander or Hispanic from pre-intervention to follow-up. Across both time points, proportions of Pacific Islander clients exceeded population estimates and Hispanic clients matched population estimates (1.5% and 16.1%, respectively) from the 2-county area (6,7).

A strength of this study is the descriptive data presented in Table 2 and Table 3, which characterize nutrient information from approximately 8.2 million calories of distributed food, presented by number of people served and by 2,000-calorie DRV. The data presented here demonstrate the need for and the potential of pantry-level PSE interventions to improve distribution of FFVs and nutritional quality of food in pantries. This study’s findings are consistent with a small but growing group of studies identifying promising approaches to improving pantry clients’ dietary quality or biometric indicators (12–14).

Food pantries serve approximately 5% of all US households per year (1). In that context, the PSE intervention’s association with increased distribution of FFVs, increased calories, and reduced sodium per 2,000 calories was a success. However, to amplify effects of pantry-level efforts to improve clients’ health, PSE interventions will likely require additional venues, including food banks, from which pantries purchase much of the food they distribute (15).
